# The Impact of Potentially Inappropriate Medications and Polypharmacy on 3-Month Hospital Readmission among Older Patients: A Retrospective Cohort Study from Malaysia

**DOI:** 10.3390/geriatrics8030049

**Published:** 2023-04-30

**Authors:** Muhammad Eid Akkawi, Hani Hazirah Abd Aziz, Abdul Rahman Fata Nahas

**Affiliations:** 1 Department of Pharmacy Practice, Faculty of Pharmacy, International Islamic University Malaysia (IIUM), Kuantan 25150, Malaysia; 2 Quality Use of Medicines Research Group, Faculty of Pharmacy, International Islamic University Malaysia (IIUM), Kuantan 25150, Malaysia

**Keywords:** older patients, polypharmacy, potentially inappropriate medication, hospital readmission, Malaysia

## Abstract

Introduction: Potentially inappropriate medications (PIMs) use and polypharmacy are two issues that are commonly encountered among older people. They are associated with several negative outcomes including adverse drug reactions and medication-related hospitalization. There are insufficient studies regarding the impact of both PIMs and polypharmacy on hospital readmission, especially in Malaysia. Aim: To investigate the possible association between polypharmacy and prescribing PIMs at discharge and 3-month hospital readmission among older patients. Materials and method: A retrospective cohort study involved 600 patients ≥60 years discharged from the general medical wards in a Malaysian teaching hospital. The patients were divided into two equal groups: patients with or without PIMs. The main outcome was any readmission during the 3-month follow-up. The discharged medications were assessed for polypharmacy (≥five medications) and PIMs (using 2019 Beers’ criteria). Chi-square test, Mann–Whitney test, and a multiple logistic regression were conducted to study the impact of PIMs/polypharmacy on 3-month hospital readmission. Results: The median number for discharge medications were six and five for PIMs and non-PIMs patients, respectively. The most frequently prescribed PIMs was aspirin as primary prevention of cardiovascular diseases (33.43%) followed by tramadol (13.25%). The number of medications at discharge and polypharmacy status were significantly associated with PIMs use. Overall, 152 (25.3%) patients were re-admitted. Polypharmacy and PIMs at discharge did not significantly impact the hospital readmission. After applying the logistic regression, only male gender was a predictor for 3-month hospital readmission (OR: 2.07, 95% CI: 1.022–4.225). Conclusion: About one-quarter of the patients were admitted again within three months of discharge. PIMs and polypharmacy were not significantly associated with 3-month hospital readmissions while male gender was found to be an independent risk factor for readmission.

## 1. Introduction

According to the World Health Organization (WHO), people are living longer globally. The percentage of older people (≥60 years old) worldwide is expected to increase from 12% to 22% between 2015 and 2050. As in Malaysia, the number of older adults is expected to increase from two million (5%) in 2010 to over six million (14.5%) by 2040 [1]. Therefore, all countries need to prepare their social and health systems to accommodate this demographic transformation.

Compared to those in middle age, older adults are more than twice as likely to need hospitalization caused by age-related reduction in physiologic reserves and higher prevalence of chronic diseases as well as drug-related hospitalization (DRH) due to drug–drug interactions, adverse drug reactions (ADRs), overdosage, or drug–disease interactions [2]. 

A high hospital readmission rate among older population might be due to many factors. One of them is the individual’s multiple diseases progression as well as deterioration of their health status that make them more prone to seek treatment [3]. Other than that, demographic factors were also found to significantly contribute to readmission. For example, male gender was associated with higher readmission risks and frequency compared to female gender in older adults [3]. Additionally, a study from the United States found that patients who were discharged without getting medical consultation or unprepared for discharge also tend to be re-admitted to the hospital [4]. 

Older patients usually need numerous medications to control their multiple diseases. Polypharmacy in older adults is well known to be associated with negative outcomes including increased ADRs and DRH [5]. Several studies have also reported polypharmacy in older adults being a risk factor for hospital readmissions [3,6,7]. On top of that, polypharmacy increases the risk of prescribing potentially inappropriate medications (PIMs) [8,9]. 

PIMs are medications that carry more risks than benefits when used in older adults as there are safer and effective alternatives [10]. Regardless of the plethora of studies in the literature warning about the negative outcomes associated with PIMs, prescribing these medications among older adults is still prevalent worldwide [11], including Malaysia [12]. Hospital admission theoretically provides a good incentive for healthcare providers to deprescribe PIMs for hospitalized patients. Nevertheless, prescribing PIMs is highly reported among discharged older adults in Malaysia [13,14]. PIMs had been linked to adverse health outcomes including ADRs, increased healthcare expenses, and DRH [15]. However, there is inconclusive evidence about the impact of PIMs on hospital readmission especially within a short period of time after discharge. Thomas et al. found a correlation between discharging patients with PIMs and hospital readmission within six months of the index admission [7], while other studies found no correlation between discharging with PIMs and hospital readmission within three months [16,17]. The objective of the study is to investigate the possible association between polypharmacy or prescribing PIMs (based on Beers’ criteria 2019) at discharge and 3-month hospital readmission among older population in Pahang, Malaysia.

## 2. Methodology

### 2.1. Setting

This retrospective cohort study was conducted at Sultan Ahmad Shah Medical Center (SASMEC), which is a teaching hospital in Kuantan, Pahang state, the largest state in the peninsular Malaysia. SASMEC provides medical services for 500,000 people living in Kuantan. The study involved older patients (≥60 years old) who were admitted to the hospital between January 2022 and September 2022.

### 2.2. Design and Study Population

Older patients who were discharged with at least one medication were included in the study. Meanwhile, patients who were (1) deceased during the initial days of the admission, (2) planned for readmission by the physicians and (3) discharged without any medication were excluded. The patients were divided into two groups i.e., study and comparison group. The study group included patients who were discharged with PIMs while the comparison group consisted of patients who were discharged without any PIMs. Both groups were followed up for three months after their discharge, where the readmission status for both groups was tracked.

### 2.3. Sample Size Calculation

The sample size for this study was estimated using the G*power software, version 3.1.9.7. The value for the proportion of group one and group two were 0.72 and 0.60, respectively, which were estimated based on a previous study [18]. A two-tailed test was used with alpha error probability values of 0.05 and 0.8 as the power of the study. As a result, the recommended sample size for each group was 241 patients.

### 2.4. Data Collection

The data were collected from the patients’ electronic charts. The wards’ records were checked on monthly basis looking for eligible patients. A comparable number of patients were included in each arm of the study every month. Demographic information, past medical history, dates of admission and discharge, list of pre-hospitalization medications, serum creatinine levels, new diagnoses, and details of discharge medications were collected. The recorded discharge medications included oral, parenteral, and inhaled medications, whereas topical medications such as eye drops and ointment were not counted. Medications that were prescribed in different doses but have the same active ingredient were considered as one medication, including obvious duplicates.

### 2.5. Data Measurement

As for polypharmacy, there is no standard definition that is being used worldwide currently. However, we considered the most frequently used definition which is the concomitant use of five or more medications [19]. Meanwhile, hyperpolypharmacy in the current study refers to the use of ten or more medications concurrently [20]. Therefore, the patients were grouped (based on the number of discharge medications) into three different categories: non-polypharmacy, polypharmacy, and hyperpolypharmacy groups. Besides, the age-adjusted Charlson comorbidity index (CCI) [21] was also utilized to assess the patients’ underlying comorbidities plus the updated list of diseases at discharge. Creatinine clearance (ClCr) was calculated using Cockcroft Gault’s formula for non-obese patients and Salazar Corcoran’s formula for obese patients (patients with body mass index ≥30). The discharge medications of the index admission were reviewed to identify PIMs using the updated Beers’ criteria 2019 of American Geriatric Society [22]. There are five types of PIMs according to the 2019 Beers’ criteria that were applied: (1) Medications that should be avoided in all older adults; (2) medications that should be avoided due to drug–disease or drug–syndrome interactions; (3) medications that should be used with caution; (4) medications that should not be used with other commonly used medications (drug–drug interactions); (5) medications that should be avoided or dose-adjusted due to renal impairment.

### 2.6. Outcome Measures

The target outcome of this study was the 3-month hospital readmission among older patients. Thus, the patients were followed up for three months after the discharge. To assess the risk factors associated with hospital readmission, a binomial logistic regression model was applied. The presence of PIMs, the presence of polypharmacy, age, gender, race, number of discharge medications, duration of the hospital stay, and AC-CCI were included in the model. Malay race and female gender were used as references for race and sex, respectively. As serum creatinine levels were not available for all patients, creatinine clearance was not included in the logistic regression model.

### 2.7. Statistical Analysis

Mean (SD) and median (IQR) were used to describe patients’ features and results. Shapiro–Wilk normality test was conducted to test the normality of continuous variables and to select the statistical tests subsequently. Pearson’s chi-squared test was used to compare the distribution of categorical variables between the control and study groups. Mann–Whitney U-test was used to compare the nonparametric variables between the two groups. Apart from that, to study the association between PIM/polypharmacy (independent variables) with hospital readmission while controlling for other variables, a binomial logistic regression model was applied as detailed above. Chi-square test was used to evaluate the significance of the model. All assumptions required to apply the binomial regression were met before running the model. The data were keyed into a Microsoft Excel sheet. The statistical analysis was conducted using the IBM SPSS Software version 27, considering *p* value of <0.05 as significant and confidence interval (CI) of 95%.

## 3. Results

### 3.1. Characteristics of the Study Population

Out of 1125 older patients who were admitted during the study period, 600 patients were included in this study after filtering for duplicates, invalid data and exclusion criteria. The study included two equal groups (300 patients/each) based on the presence of PIMs at discharge. The median age for PIMs and non-PIMs patients were 70 and 69 years old, respectively. Meanwhile, the proportion for Malay and non-Malay patients were the same for both groups, which were 88.67% and 11.33%, respectively. Other than that, male patients dominated over female patients for both PIMs and non-PIMs groups. Plus, both groups had comparable duration of hospital stay, which was about 5–6 days (Table 1).

### 3.2. Patients’ Medical Profiles

The most common diseases at discharge were hypertension and diabetes mellitus. The median CCI scores for both PIMs and non-PIMs patients was five. Serum creatinine readings were available for 361 patients. The prevalence rates of patients with ClCr < 60 mL/min were comparable in the two groups of the study (Table 1).

At discharge, 3513 medications were dispensed with median (IQR) number of medications of 6 (4) medications per patient. The most common discharge medications were antiplatelet agents (used in 60.5% of the patients), statins (54%), proton pump inhibitors (46.33%), beta adrenergic blockers (33.67%), and insulin preparations (28.33%). Patients who were on PIMs at discharge had a higher number of medications compared with the non-PIMs group. Overall, 65.1% of the patients were discharged on polypharmacy (55.8%) and 9.3% on hyperpolypharmacy. The prevalence of polypharmacy was also higher among PIMs patients than that in the non-PIMs group (Table 1).

### 3.3. PIMs Use

Out of the 300 patients in the study group, 260 patients (86.7%) were discharged with one type of PIMs, 33 patients (11%) with two types of PIMs while only 7 patients (2.4%) with three types of PIMs; resulting in a total of 347 PIMs prescriptions at discharge. The most frequently prescribed PIMs were medications that should be used with caution in older adults (59.65%), followed by medications that should be avoided in all older adults (27.09%); medications that should be avoided or dose-adjusted due to renal impairment (11.82%); medications that should not be used with other commonly used medications due to drug–drug interactions (1.15%); and medications that should be avoided due to drug–disease or drug–syndrome interactions (0.29%). 

The list of medications identified as PIMs were presented in Table 2. There was a total of 28 types of medications prescribed as PIMs during the discharge. The most commonly recognized medications that should be avoided in all older adults was peripheral alpha-1 blockers (7.2%) for the treatment of hypertension in patients without concomitant prostate hyperplasia, which increases the risk for orthostatic hypotension and related harms especially in older adults. There was only one medication identified as medication that should be avoided due to drug–disease or drug–syndrome interactions, which was the use of celecoxib (0.28%) in patients with symptomatic heart failure as it could cause fluid retention and/or worsen heart failure. The most common medication that should be used with caution was aspirin (33.43%) as primary prevention of cardiovascular disease, which could increase the risk of major bleeding. Medications that should be avoided due to drug–drug interactions were not common as they were found in four patients only. Lastly, rivaroxaban (3.46%) was the most frequently prescribed medications that should be avoided or dose-adjusted due to renal impairment, which was given to twelve patients with ClCr < 50 mL/min (Table 2).

### 3.4. Hospital Readmission within Three Months and Its Risk Factors

Of the 600 patients included in the study, 152 (25.3%) patients were re-admitted within three months of their discharge. None of the patients’ characteristics were significantly associated with the readmission except the gender. More male patients (26.7%) were rehospitalized within three months than female patients (14.7%). The prevalence of 3-month hospital readmission in the study group (25%) was analogous to that in the comparison group (25.7%). Additionally, being discharged with polypharmacy/hyperpolypharmacy was not associated with hospital readmission (Table 3).

Applying binomial logistic regression confirmed that only gender was a significant predictor of hospital readmission. The risk of hospital readmission was 2.07 times higher (95% CI: 1.022–4.225) in male than female patients. All other variables included in the regression model did not significantly predict the incidence of hospital readmission within three months. The applied logistic regression model statistically and significantly predicted hospital readmission, χ^2^ (9) = 18.797, *p* = 0.027.

## 4. Discussion

This study investigated the impact of PIMs and polypharmacy on 3-month hospital readmission among older patients. Our findings showed that more than half of the studied sample (65.1%) were discharged with five or more medications (polypharmacy). The high prevalence of polypharmacy among discharged older patients had been reported by another study from Pahang, Malaysia, where the prevalence of polypharmacy was 66.3% at discharge [14]. Similar findings were declared from other countries [23,24]. However, hyperpolypharmacy was more prevalent at discharge in other studies than in ours [25,26]. Polypharmacy is known to be associated with various negative outcomes including declined physical, cognitive, and emotional capabilities as well as hospitalization [5,27]. Several studies have correlated polypharmacy with hospital readmission among older patients [3,6,7]. However, this was not the case in our study, in which neither polypharmacy nor hyperpolypharmacy was significantly related to hospital readmission. It is logical that prescribing numerous medications increases the risk for one of them to be inappropriate. Many studies have reported polypharmacy as an independent risk factor for prescribing PIMs [19,20,28]. Our study also demonstrated that the number of prescribed medications together with polypharmacy status had a significant association with the incidence of PIMs prescribing. The result was consistent with studies conducted in Malaysia [29,30]. In most of the cases, polypharmacy is inevitable in older patients due to the high number of diseases. Therefore, contemporary studies focus on the appropriate use of polypharmacy among older patients rather than just the number of medications [31].

PIMs use in older adults should be avoided as it was evident that they cause increased risk of adverse drug events, high healthcare expenses, disability, and worsened self-reported health and death [30]. There are several tools that can be used to detect PIMs, with Beers’ criteria being one of the most common tools used in the literature (Ma et al., 2018; Tao et al., 2021). After applying Beers’ criteria 2019 in our study, the most frequently encountered PIMs were medications that should be used with caution, namely aspirin for primary prevention of CVDs and tramadol. These results are not parallel with many of other studies where the use of proton pump inhibitor (PPI) for more than eight weeks, vasodilators, and benzodiazepines (BZDs) were the predominant PIMs reported from Malaysia and other countries [2,16,18,32,33]. However, a study in China found that medications to be used with caution were the most common PIMs. Nevertheless, spironolactone and furosemide were the predominant PIMs [20]. One study from Australia reported findings similar to ours where aspirin for primary prevention of CVD was the second top prescribed PIMs [8]. The differences in the findings might be due to the variances of the study population’s characteristics. In other words, different populations have different demographic backgrounds, common comorbidities, and medical practices according to the country which in turn affects the common medications to be prescribed. As mentioned earlier, PIMs are associated with different noxious effects. Thus, it is crucial to review older patients’ medications regularly and deprescribe unnecessary and inappropriate medications to reduce polypharmacy and PIMs and their consequences [34]. However, several barriers to deprescribing were identified in the literature. This includes inadequate knowledge of physicians about misprescribing/overprescribing, poor coordination between prescribers, patient’s refusal to change their medications, and lack of time to check for the appropriateness of the prescribed medications [35].

Our study found that prescribing PIMs at discharge did not have a significant contribution to 3-month readmissions. This result contradicts what has been reported in the literature, where PIMs increased the risk of hospital readmission [7,18,28]. One of the possible explanations for this difference is the duration of follow-up. The above-mentioned studies followed up discharged patients for six months [7] or even 1–4 years [18,28]. The results from studies that limited the duration of follow-up to three months or shorter were consistent with our findings [6,16,17]. Other than the difference in the follow-up duration, the types of prescribed PIMs might contribute to the difference in the outcomes. For instance, prescribing BZDs was common in the other studies [18,36] but not in ours. Using BZDs was reported as risk factor for hospitalization as they increase the risk for falls in older patients [37,38]. It can be concluded that evidence about the impact of PIMs on short-term hospital readmission is still limited and inconclusive. However, this surely does not imply the safety of using PIMs among discharged older patients. 

The only risk factor that was associated with 3-month hospital readmission was male gender. The logistic regression model showed that male gender was a predictor for readmission after controlling for all other variables. This result agrees with the findings from other studies where male gender was an independent factor for short-term hospital readmission [3,7,16]. This gender difference in the rate of hospitalization could be attributed to the fact that women are generally more aware of health preventive measures and more eager in seeking medical care than men [39]. Other than that, length of hospital stay was not correlated to 3-month readmission among our patients, which is consistent with the Japanese study that followed the patients for up to three months [16]. Likewise, CCI did not significantly affect 3-month hospital readmission in our study. Nonetheless, some studies described it as one of the risk factors of readmission [6,16].

## 5. Limitations and Strengths

First of all, the results presented in this study were only representing older adults in Kuantan, Pahang, and, therefore, it might not be applicable and generalized to all health care settings in Malaysia. Another limitation is the lack of some information that could be attributed to identifying PIMs. For example, creatinine clearance could not be calculated for all patients due to unavailability of serum creatinine readings. Moreover, as it is a retrospective study, it did not include several detailed clinical parameters among the study population such as geriatric depression scale (GDS), mini mental state examination (MMSE), and basic activities of daily living (BADL), which might be other risk factors for hospital readmissions. Lastly, we did not assess the medication adherence among the study population. Thus, we are unsure if the patients had poor adherence, which might contribute to hospital readmissions.

As for the strengths of our study, we used the most updated Beers’ criteria to assess PIMs use, which was published in 2019. This study also provided confounding factors for rehospitalization other than medication-related factors. Plus, the sample size used in this study could also be considered as sufficient to represent overall geriatric patients in the state of Pahang as SASMEC is a referring hospital for the whole state. 

## 6. Conclusions

The number of medications at discharge as well as polypharmacy status were found as the main reason for the use of PIMs. Medications that should be used with caution were the most common type of PIMs prescribed at discharge. Hospital readmission within three months of discharge was recorded in one-quarter of the patients. Discharge PIMs and polypharmacy did not increase the risk for the readmission. Male gender was found to be the only predictor of 3-month readmission. A larger prospective study is recommended to identify the impact of PIMs on hospital readmission and the strategies to reduce the use of PIMs in this population.

## Figures and Tables

**Table 1:** Demographic and medical characteristics of the studied patients (n: 600) divided according to PIMs and non-PIMs use at discharge.

Variables	Patients Discharged with PIMs (n: 300)	Patients Discharged without PIMs (n: 300)	*p* Value
Age, (median [IQR])	70 (10)	69 (10)	0.074
Race, n (%)			
Malay	266 (88.67)	266 (88.67)	1.000
Other races	34 (11.33)	34 (11.33)	
Gender, n (%)			
Male	265 (88.33)	267 (89)	0.797
Female	35 (11.67)	33 (11)	
Comorbidities, n (%)			
Hypertension	225 (75)	204 (68)	
Diabetes Mellitus	164 (54.67)	153 (51)	
Dyslipidemia	117 (39)	111 (37)	
Chronic kidney disease	60 (20)	51 (17)	
CCI (median [IQR])	5 (2)	5 (2)	0.443
Creatinine clearance (361 patients), (n [%])			
>60 mL/min	127 (68.3)	117 (66.9)	0.630
<60 mL/min	59 (31.7)	58 (33.1)	
Days of hospital stay (median [IQR])	5.5 (6)	5 (5)	0.223
Number of medications at discharge (median [IQR])	6 (3)	5 (4)	<0.001
Polypharmacy status, n (%)			
No polypharmacy	74 (24.7)	135 (45)	<0.001
Polypharmacy	193 (57.6)	142 (47.3)	
Hyperpolypharmacy	33 (11)	23 (7.7)	

**Table 2:** Types of the PIMs prescriptions (n: 347).

Type of PIM	Name of Medication	Prevalence, n (%)
Medications that should be avoided in all older adults	Metoclopramide	9 (2.59)
	PPI (used for >12 weeks)	25 (7.20)
	Digoxin for first-line treatment for AF	2 (0.58)
	Peripheral alpha-1 blockers for treatment of hypertension	25 (7.20)
	Sliding scale insulin	4 (1.15)
	Amitriptyline	2 (0.58)
	Medications with strong anticholinergic properties (Prochlorperazine-Trihexyphenidyl)	5 (1.47)
	Benzodiazepines	10 (2.88)
	First-generation antihistamines	11 (3.17)
	NSAIDs for chronic use	1 (0.28)
Medications that should be avoided due to drug–disease or drug–syndrome interactions	Celecoxib (used in patients with symptomatic heart failure)	1 (0.28)
Medications that should be used with caution	Rivaroxaban	39 (11.24)
	Aspirin (as a primary prevention of CVDs)	116 (33.43)
	Dabigatran	4 (1.15)
	Tramadol	46 (13.25)
	Sulfamethoxazole-trimethoprim	2 (0.58)
Medications that should not be used with other commonly used medications (drug–drug interactions)	Pregabalin-morphine	1 (0.28)
	Gabapentin-tramadol	1 (0.28)
	Ciprofloxacin-theophylline	2 (0.58)
Medications that should be avoided or dose-adjusted due to renal impairment	Apixaban	6 (1.73)
	Enoxaparin	1 (0.28)
	Spironolactone	6 (1.73)
	Levetiracetam	2 (0.58)
	Colchicine	4 (1.15)
	Rivaroxaban	12 (3.45)
	Pregabalin	3 (0.86)
	Gabapentin	1 (0.28)
	Tramadol	6 (1.73)
**Total**		**347**

PPI: proton pump inhibitor, AF: atrial fibrillation, CVDs: cardiovascular diseases, NSAID: non-steroidal anti-inflammatory drug.

**Table 3:** Prevalence of hospital readmission based on the patients’ characteristics (n: 600).

Variable	Hospital Readmission		*p* Value
	Yes n (%)	No n (%)	
Age category			
60–69 years	71 (24.7)	216 (75.3)	0.241
70–79 years	64 (27.4)	170 (72.6)	
80–89 years	12 (17.9)	55 (82.1%)	
90–99 years	5 (41.7)	7 (58.3)	
Gender			
Male	142 (26.7)	390 (73.3)	0.032
Female	10 (14.7)	58 (85.3)	
Race			
Malay	136 (25.6)	396 (74.4)	0.716
Non-Malay	16 (23.5)	52 (76.5)	
Polypharmacy status at discharge			
No polypharmacy	48 (23)	161 (77)	0.08
Polypharmacy	83 (24.8)	252 (75.2)	
Hyperpolypharmacy	21 (37.5)	35 (62.5)	
Having PIMs at discharge			
Yes	75 (25)	225 (75)	0.851
No	77 (25.7)	223 (74.3)	
ClCr level *			
<60 mL/min	74 (30.3)	170 (69.7)	0.124
>60 mL/min	45 (38.5)	72 (61.5)	

* This includes 361 patients.

## Data Availability

Data are available upon a request from the corresponding authors.
